# Effects of Structural Changes in Cross-Linked Mung Bean Starch on Freeze–Thaw Properties and In Vitro Digestibility

**DOI:** 10.3390/foods14040689

**Published:** 2025-02-17

**Authors:** Altantungalag Agvaandorj, Yuanzao Li, Junhee No

**Affiliations:** 1 Department of Food Science and Nutrition, Kyungpook National University, Daegu 41566, Republic of Korea; tungalag0302@knu.ac.kr (A.A.); lyz2024@knu.ac.kr (Y.L.); 2 The Center for Beautiful Aging, Kyungpook National University, Daegu 41566, Republic of Korea

**Keywords:** mung bean starch, cross-linked starch, resistant starch, RS4, in vitro digestibility, freeze–thaw stability

## Abstract

This study aims to evaluate the effects of structural changes in cross-linked mung bean starch (CLMB) on freeze–thaw stability and in vitro digestibility and explore its potential to prevent starch retrogradation and its applicability as a resistant starch (RS)- enhanced food ingredient. Mung beans of different varieties (Eohul, Geumsung, and Sohyeon) were cross-linked using an STMP:STPP ratio of 9:1. The structure and thermal properties of CLMB and its digestibility, as well as the textural properties of 10% CLMB gels and their freeze–thaw stability, were evaluated. As a result of the study, CLMB maintained an A-type crystalline structure, but structural changes due to the introduction of phosphate groups were observed during FT-IR analysis. Compared to natural mung bean starch (MBS), the swelling power and solubility decreased, and the gelatinization temperature range increased. Additionally, the cross-linking treatment increased the resistant starch (RS) content. In the case of the gel with 10% CLMB added, the freezing–thawing experiment results show a significant reduction in syneresis and it was confirmed that high stability was maintained even through repeated processes. Our results suggest that CLMB is a functional ingredient with potential applications in the development of food products offering extended shelf lives and tailored nutritional benefits.

## 1. Introduction

Mung bean (*Vigna radiate* L.; MB) has been cultivated in Asia since ancient times and is now widely grown in Africa, South America, Australia, and the United States [1]. Its seeds contain approximately 31–42% starch, making them an exceptional source of carbohydrates compared to other leguminous crops [1,2]. The starch in MBs is distinguished by its high amylose content (25–30%) and remarkable gel stability [2,3]. Compared to starches from corn, rice, potatoes, and other sources, mung bean starch presents unique characteristics due to its high viscosity, tendency to undergo retrogradation, dehydration shrinkage, weak shear strength, and sensitivity to temperature and pH fluctuations [1,3,4,5]. These characteristics can significantly limit its industrial applications, particularly in food products that require stable textures, smoothness, and resistance to mechanical stress [1,4,5]. The high viscosity of mung bean starch has been reported to reduce its applicability in products that demand a smoother texture [6]. The tendency for retrogradation and dehydration shrinkage further renders mung bean starch unsuitable for products where consistency and long-term stability are essential [7]. Additionally, its weak shear strength and instability under fluctuating temperature and pH conditions further limit its use in applications such as sauces, soups, and processed meals, where starches from maize, rice, and potatoes are more dependable [3].

Starch modification is considered a solution to overcome the limitations of native starches, and research on the physical, chemical, and enzymatic modification of MB starch has been conducted. The chemical modification of native starch involves the addition of carboxyl, acetyl, or phosphate groups through processes such as esterification, etherification, and cross-linking [4]. Such modifications have primarily focused on altering the properties and structural integrity of starch granules to enhance characteristics such as gelatinization and reduce retrogradation [8].

Cross-linking represents a widely employed chemical modification technique for enhancing the functional properties of starch. This process involves forming ether or ester linkages between the hydroxyl groups of native starch molecules using a cross-linking agent [9]. Sodium trimetaphosphate (STMP) and sodium tripolyphosphate (STPP) are among the most commonly used cross-linkers in the food industry due to their cost-effectiveness, supply stability, and non-toxic nature [10]. In the United States, the allowable residual phosphorus content in processed foods after the use of STMP and STPP is 0.4% [8]. Cross-linking and phosphorylation reactions caused by STMP/STPP are generally observed in the amorphous regions of starch granules [4,11]. Therefore, the structure of starch may influence cross-linking. The gel strength and viscosity of STMP/STPP cross-linked starches are significantly enhanced, even at low levels of phosphorylation. Additionally, their water retention capacities are higher compared to native starches, and the structural integrity of granules is retained, leading to improved resistance to retrogradation [8,12]. According to Ashwar et al. [4], phosphorylation and cross-linking have been very useful in reducing retrogradation and improving the thermal stability of starch, addressing the critical challenges faced by native starches in industrial food applications. Thus, STMP/STPP cross-linking can be expected to mitigate the limitations of rapid retrogradation in MB starch.

Most previous studies on cross-linked starches have focused on more common starch sources, such as rice, maize, potato, and tapioca. According to a study by Sha et al. [10], adding STMP/STPP cross-linked wheat starch to fried noodles reduced cooking loss, increased thermal resistance, and enabled the development of foods with lower starch digestibility. Additionally, cross-linked starch molecules have been reported to provide high resistance to digestive enzymes, leading to an increase in resistant starch content [7,10]. Studies on gluten-free bread made with phosphorylated corn starch have reported a reduction in bread hardness and improved freeze–thaw stability [11]. This suggests that starch modified through cross-linking with STMP/STPP can serve as a functional additive, increasing resistant starch content via structural alterations and enhancing the texture of food products. Therefore, cross-linking treatment contributes to inhibiting starch retrogradation while enhancing resistant starch content, which supports the development of health-oriented foods for glycemic response regulation.

Additionally, cross-linked potato, banana, and corn starches prepared with STMP/STPP effectively formed complexes with C-phycocyanin, demonstrating potential use as a wall material in prolonged-release pharmaceutical formulations [12], and tapioca starch cross-linked with STMP was observed to preserve its structure and demonstrated improved freeze–thawing stability, making it suitable for use as a bio-film material [9]. Therefore, cross-linked MB starch not only compensates for the limitations of its use in the food industry but also suggests potential applications in emerging material industries such as biofilms.

However, studies analyzing the structures of starches from different MB varieties or the correlations between the structural changes induced by cross-linking and the physicochemical properties of these varieties’ starches are limited. In particular, we know of no study examining changes in freeze–thaw stability and digestibility due to structural modifications of MB starch, highlighting a need for research to enhance the usability of MB starch.

In this study, MB starch from different varieties was cross-linked using STMP/STPP, and the resulting product’s structural properties, in vitro digestibility, gel stability, and freeze–thaw stability were evaluated. This study aimed to investigate the effects of cross-linking on the structural changes of MB starch and analyze the correlations between these modifications and in vitro digestibility, gel texture, and freeze–thaw stability. The findings of this study are expected to provide fundamental insights into the increased resistant starch content and altered physicochemical properties of cross-linked mung bean starch, serving as a basis for its potential applications in various food and material industries.

## 2. Materials and Methods

### 2.1. Materials

Mung bean seeds of three different Korean varieties—Eohul (E), Geumsung (G), and Sohyeon (S)—were obtained from the Rice Institute of Jeonnam Agricultural Research & Extension Services (Nampyeong, Republic of Korea). Pancreatin from porcine pancreas (P7545, 8 × USP specifications), STMP (≥95%), STPP (85%), and amyloglucosidase (A9913) were purchased from Merck KGaA (Darmstadt, Germany), and GOPOD (glucose oxidase/peroxidase) kits were purchased from Megazyme International Ireland Ltd. (Wicklow, Ireland). Ethanol (99.9%), sodium hydroxide (NaOH, 93%), hydrochloric acid (HCl, 35%), sodium sulfate (Na_2_SO_4_, 99%), and sodium acetate (98.5%) were purchased from Duksan Pure Chemicals Co., Ltd. (Ansan, Republic of Korea).

### 2.2. Isolation of Native MB Starch

The isolation of native MB starch (NMB) was carried out following the procedure outlined by No and Shin [13]. The MB seeds were soaked, dehulled, and ground. The starch was isolated by sieving (100- and 200-mesh) and centrifuging with distilled water. The starch precipitates were then dried, ground, and passed through a 100-mesh sieve.

### 2.3. Preparation of Cross-Linked MB Starches

The preparation of cross-linked MB starches was carried out following the procedure outlined by Song et al. [14]. First, NMB and deionized water were combined in a 1:1 (*w*/*v*) ratio and subjected to annealing in a 50 °C water bath with constant stirring at 80 rpm for 12 h. Following the annealing process, 10% Na_2_SO_4_, 11.98% STMP, and 0.02% STPP were added. The pH of the solution was then brought to 11.5 using 1 N NaOH, and the cross-linking reaction was carried out at 45 °C in a water bath with continuous stirring for 3 h. Following the reaction, the pH was adjusted to neutrality (7.0) using 1 N HCl. The sample was repeatedly washed using deionized water via centrifugation (Supra 22 K, Hanil Science Industrial Co., Gimpo, Republic of Korea) at 1100× *g* for 10 min, dried at room temperature, and sieved through a 100-mesh sieve.

### 2.4. Measurement of the NMB Molecular Weights

The molecular weights of the NMB from each MB variety were measured using high-performance size exclusion chromatography (HPSEC 1100 series, Agilent Technologies, Santa Clara, CA, USA) with a refractive index detector (Shodex RI-101, Showa Denko, Tokyo, Japan), following the method described by Oh and Shin [15]. Shodex OHpak SB-806 HQ and Shodex OHpak SB-804 HQ columns (8.0 × 300 mm each, Showa Denko, Tokyo, Japan) were used. Filtered and degassed deionized water was used as the mobile phase, with a flow rate of 0.6 mL/min. Shodex standard P-82 was used for calibration. The molecular mass was measured using a GPC ELEOS System (Wyatt Technologies Inc., Goleta, CA, USA).

### 2.5. Measurement of Amylopectin Branch Chain Distributions in NMB

The amylopectin branch chain distributions of the NMBs were analyzed using high-performance anion exchange chromatography with a pulsed amperometric detector (HPAEC-PAD, Dionex, Sunnyvale, CA, USA), following the method described by Oh and Shin [15]. A filtered starch solution was injected into a Dionex CarboPac PA-200 column (250 × 3 mm) with a flow rate of 0.5 mL/min. The starch solution was eluted with a sodium acetate/sodium hydroxide gradient, prepared using 150 mM sodium hydroxide (eluent A) and a 500 mM sodium acetate and 150 mM sodium hydroxide solution (eluent B). The amylopectin branch chain-length distribution was calculated based on relative peak areas up to a degree of polymerization (DP) of 60.

### 2.6. Morphology Measurement

The NMB and cross-linked MB starch (CLMB) samples were attached to an SEM stub and then coated with Pt/Au following the method described by No and Shin [16]. The samples were examined under a scanning electron microscope (SEM; SU8220, Hitachi High-Technologies, Tokyo, Japan) at 15 kV and ×1000 magnification.

### 2.7. Crystalline Structure Measurement

The degrees of relative crystallinity and crystallinity of the NMBs and CLMBs were analyzed using an X-ray diffractometer (Empyrean, Malvern Panalytical, Almeo, The Netherlands) targeting Cu-Kα with a Ni filter, the voltage set to 40 kV, and the current set to 30 mA. The samples were scanned at 2θ values from 5 to 40°. The relative crystallinity was calculated using Origin 2024 (Microcal, Northampton, MA, USA) following the method described by No and Shin [16].

### 2.8. Fourier Transform Infrared Spectroscopy

Fourier transform infrared (FT-IR) spectrometer (Frontier, PerkinElmer, Waltham, MA, USA) was used to measure the phosphorylation of the NMBs and CLMBs based on the attenuated total reflectance technique. The spectrum was collected over a 4000–400 cm^−1^ frequency range at a resolution of 4 cm^−1^ using Spectrum™ 10 software (PerkinElmer, Waltham, MA, USA) and measured at least twice [4].

### 2.9. Water-Binding Capacity Measurement

The NMB and CLMB (0.5 g, dry basis) samples were mixed with 20 mL of distilled water for 1 h at room temperature. The samples were then centrifuged, and the sediment was collected and weighed. The water-binding capacity (WBC) was calculated using the equation [17]:

WBC (%) = (sediment weight (g) − sample weight (g))/sample weight (g)

### 2.10. Measurement of Swelling Power and Solubility

The NMB and CLMB (0.2 g, dry basis) samples were mixed with 20 mL of distilled water and stirred using a magnetic bar for 30 min in an 80 °C water bath. After cooling and centrifugation, the collected supernatant was dried at 105 °C to determine solubility (% by weight), and the residue was weighed to determine the swelling power (g/g) [17]:

Solubility (%) = dried supernatant weight (g)/sample weight (g) × 100

Swelling power (g/g) = weight of swollen sample weight (g)/[sample weight (g) × (100 − solubility (%))]

### 2.11. Differential Scanning Calorimetry

The thermal properties of the NMBs and CLMBs were determined using differential scanning calorimetry (DSC; DSC-Q1000, Universal V.3.6C TA Instruments, Olivia Gibson, New Castle, UK). The NMB and CLMB (3.0 mg, dry basis) samples were weighed in an aluminum pan (aluminum pan/lid set (900793.901/900794.901, TA Instruments), and 6.0 mg of distilled water was added. The pan was sealed and kept overnight at room temperature to stabilize. Then, the sample was heated from 30 to 130 °C at a heating rate of 10 °C/min.

### 2.12. Measurement of In Vitro Digestibility

The in vitro digestibility of the NMBs and CLMBs was analyzed using the method described by No and Shin [16]. A 200 mg sample was mixed with phosphate buffer (pH 5.8) and incubated with porcine pancreatic α-amylase and amyloglucosidase for 20 (G20) and 120 min (G120), respectively, in a 37 °C water bath. The glucose content in the supernatant was measured using the GOPOD method to calculate the rapidly digestible starch (RDS), slowly digestible starch (SDS), and resistant starch (RS). The digestibility (%) was expressed as g/100 g of starch and determined based on the amount of glucose released into the supernatant. The glucose concentration was multiplied by 0.9 to estimate the mass of digested starch. RDS was hydrolyzed within 20 min, while SDS was digested within 120 min. RS refers to the fraction that remained unhydrolyzed beyond this period.

### 2.13. Measurement of the Gel Properties and Freeze–Thaw Properties of CLMBs

#### 2.13.1. Preparation of NMB and CLMB Gels

Starch gels were prepared to evaluate the gel properties and freeze–thaw stabilities of the NMBs and CLMBs with modifications based on the methods No and Shin [13]. Since CLMB cannot form a gel on its own, gels were prepared using 9:1 NMB-CLMB mixtures and compared with the corresponding NMB gel. Three grams of the mixture was suspended in 25 mL of distilled water, and the suspension was gelatinized in a boiling water bath for 5.5 min with a magnetic stirring. After heating, the starch pastes were poured into a 1.5 × 1.5 cm stainless steel container and cooled at room temperature for 6 h.

#### 2.13.2. Measurement of Hunter L, a, and b Values

The color of the gels was analyzed using a spectrocolorimeter (Lovibond LC 100, Tintometer GmbH, Dortmund, Germany). Three values, L (lightness), ±a (redness/greenness), and ±b (yellowness/blueness), were recorded for each starch gel, and the ΔE value against a white reference (L = 96.81, a = −0.09, and b = −0.18) was calculated.

#### 2.13.3. Measurement of Surface Appearance

To assess surface appearance characteristics, the NMB and CLMB gels were placed on black paper and photographed using a camera (iPhone 15 pro, Apple Inc., Cupertino, CA, USA) at a distance of 10 cm.

#### 2.13.4. Textural Properties Measurement

The texture properties of NMB and CLMB gels were analyzed using a texture analyzer (CT3, Brookfield Ametek, Middleborough, MA, USA) through two-bite compression tests (texture profile analysis, TPA). The NMB and CLMB gels were both measured immediately after preparation (fresh gels) and refrigeration for 16 h. The TPAs were performed using a sample size of 1.5 × 1.5 cm; cylinder-type probe (Φ 20 mm); a deformation rate of 60%; and pre-test, test, and post-test speeds of 1 mm/s. Based on the resulting TPA curve, gel hardness, cohesiveness, springiness, gumminess, and resilience were determined.

#### 2.13.5. Measurement of Freeze–Thaw Stability of NMB and CLMB Gels

The freeze–thaw stability of the NMB and CLMB gels was evaluated following the method outlined by Park et al. [18]. Gels were transferred into tubes and frozen at −20 °C for 22 h before being thawed at room temperature for 2 h. This freeze–thaw cycle was repeated up to five times. Then, the gels were centrifuged at 1310× *g* for 10 min, and the amount of water separated from the gels was measured to determine syneresis.

### 2.14. Statistical Analysis

The experimental results are presented as means ± standard deviations from three or more experiments. Data were analyzed using analyses of variance (ANOVAs) followed by Duncan’s multiple range tests, with *p* < 0.05 accepted as indicating statistical significance. Tests were performed with IBM SPSS Statistics for Windows version 27 (IBM Corp., Armonk, NY, USA).

A principal component analysis (PCA) was conducted using Origin 2024 (Microcal, Northampton, MA, USA) to analyze and visualize the relationships between the NMBs and CLMBs and their characteristics.

## 3. Results and Discussion

### 3.1. Molecular and Physicochemical Properties of NMB and CLMB

#### 3.1.1. Molecular Weight Distributions and Apparent Amylose Content of NMBs

**Figure 1:**
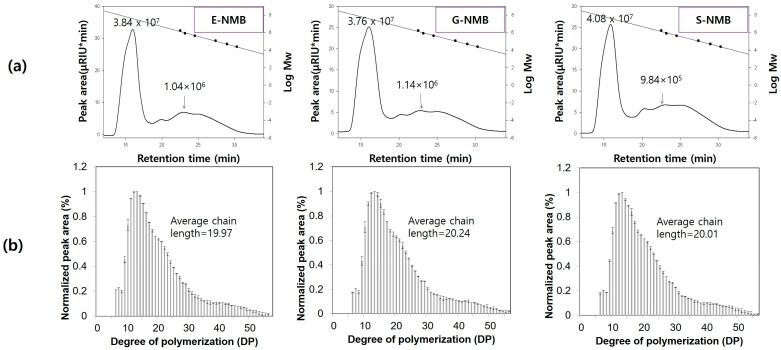
Molecular weight distributions (**a**) and amylopectin branch chain distributions (**b**) of native mung bean starches from the Eohul (E-NMB), Geumsung (G-NMB), and Sohyeon (S-NMB) varieties.

The molecular weight distribution and apparent amylose contents of the NMBs are shown in Figure 1. Two distinct peaks were observed in the molecular weight profile. Generally, larger molecules elute earlier than smaller molecules, and under the separation conditions, a sharp amylopectin peak at a retention time of approximately 15 min was followed by a broad amylose peak between 20 and 30 min, as reported previously [19]. The molecular weight of amylopectin was 3.84 × 10^7^ g/mol for E-NMB, 3.76 × 10^7^ g/mol for G-NMB, and 4.08 × 10^7^ g/mol for S-NMB, while those of amylose were 1.04 × 10^6^, 1.14 × 10^6^, and 9.84 × 10^5^ g/mol for E-NMB, G-NMB, and S-NMB, respectively.

The molecular weight of amylopectin in NMB has previously been reported as 4.1 × 10^7^ g/mol, aligning well with our results [20]. However, in a previous study focusing on legume starches, NMB had amylopectin and amylose molecular weights of 264 × 10^6^ g/mol and 1.83 × 10^6^ g/mol, respectively, both of which are lower than the amylopectin molecular weights observed in this study [21]. Similarly, Kim et al. [22] found that amylopectin molecular weights ranged from 66.5 to 88.8 × 10^6^ g/mol, and those of amylose ranged from 1.8 to 3.0 × 10^6^ g/mol. The observed discrepancies may result from the degradation of amylopectin and amylose caused by the use of alkali during the starch isolation processes in these previous studies [20]. We isolated NMBs using distilled water, minimizing structural degradation. Variations in molecular weight distributions among different starches can also be attributed to genetic differences between cultivars, which are known to significantly influence the molecular characteristics of starch [23].

The apparent amylose contents (%) of E-NMB, G-NMB, and S-NMB were 39.72, 39.28, and 40.43%, respectively, with no statistically significant differences observed between the varieties. Previous studies have reported that legume starches have a high amylose content (30–40%) [21]. Park et al. [24] reported the apparent amylose contents of E-NMB, G-NMB, and S-NMB as 39.7%, 40.3%, and 42.2%, respectively, which is consistent with the findings of this study. Due to its high amylose content, MB starch is expected to form firm and elastic gels. In particular, previous studies have shown that amylose in NMB remains within the granules, demonstrating restricted leaching as a critical factor determining the gel’s mechanical strength and physical properties [25]. Therefore, it can be concluded that the MB cultivar significantly influences the properties of the starch, and the molecular weight and content of amylose are likely to impact its viscoelastic characteristics [23]. Additionally, differences in starch structure among cultivars may lead to variations in the characteristics of starch upon cross-linking treatment.

#### 3.1.2. Amylopectin Branch Chain Length Distributions in NMB

The amylopectin chain length distributions of the NMBs are shown in Figure 1. The average amylopectin chain length ranged from 19.97 to 20.24, with no significant differences observed among the MB cultivars. Amylopectin branch chains with degrees of polymerization (DPs) of 13–24 accounted for the highest proportion at 52.22–52.75%, whereas branch chains with DPs ≥ 37 represented the lowest proportion at 8.00–8.86% among all MB varieties. In a previous study, the distribution of amylopectin branch chain lengths in various legume starches showed similar results, with DPs of 6–12, 13–24, 25–36, and ≥37 representing 21.63–42.65, 44.71–59.45, 7.35–11.15, and 4.34–8.31%, respectively [26]. Amylopectin chains are categorized into A, B, and C chains, with B chains further divided into B1, B2, and B3 chains based on the number of clusters [22]. A chains have DPs of 6–12, B1 chains have DPs of 13–24, B2 chains have DPs of 25–36, and B3 chains have DPs ≥ 37 [16]. Additionally, amylopectin B2 and B3 chains are predominantly found in the amorphous region, while A and B1 chains are located in the crystalline region [27].

The structure of amylopectin is particularly important because phosphate groups in native starches have been reported to form covalent bonds with amylopectin [8]. Waxy corn starch has been reported to retain more phosphate salts within its branched amylopectin chains when phosphorylated using STMP, even compared to other starch types [28]. The cross-links in CLMB may form through covalent bonding between amylopectin chains. The decrease in apparent amylose content observed in phosphorylated rice starch has been attributed to the formation of intermolecular bonds between amylose molecules or amylose and amylopectin [4]. Therefore, phosphorylation is believed to influence the structure of amylose and amylopectin in starch. The formation of SDS and RS is facilitated by long amylopectin chains during the production of modified starch, and these chains may also influence the functionality of amylose [23]. Thus, since we found that the NMBs’ structures contained high proportions of long amylopectin branched chains, we expected to find enhanced resistant starch content after cross-linking with STMP/STPP.

#### 3.1.3. NMB and CLMB Morphologies

**Figure 2:**
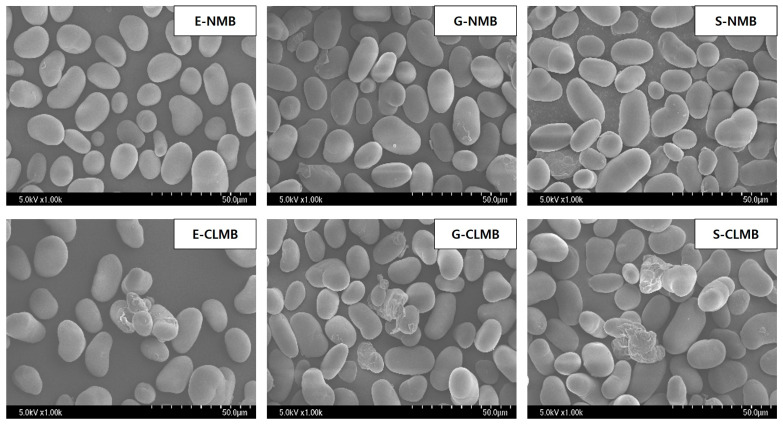
Morphology of native mung bean starches (NMBs) and cross-linked mung bean starches (CLMBs) from three mung bean varieties: Eohul (E), Geunsung (G), and Sohyeon (S).

The morphologies of the NMBs and CLMBs were examined through SEM (Figure 2). The NMBs displayed a typical bimodal granular shape, in which the large granules had an oval or elliptical shape, while the small granules exhibited a round shape. This aligns with the findings of Huang et al. [29]. The CLMBs retained their original bimodal granular shapes, indicating that the particle shape of the NMB was not significantly altered by cross-linking with STPP and STMP. However, some granules exhibited splitting, cracks, and corrosion, with small pores observed on the surface. The cross-linking of corn starch granules using the slurry method with STMP/STPP resulted in the formation of agglomerated starch granules [11], and the treatment of tapioca starch with STPP also resulted in the formation of some pores, indicating that the ordered crystalline structure in certain starch granules was disrupted [9]. However, in the present study, the structural integrity of CLMB was largely retained, indicating that the starch structure was strengthened and stabilized by cross-linking. Previous studies have also shown that phosphorylation combined with moist heat treatment minimizes structural changes in starch [29].

#### 3.1.4. Crystallinity of NMBs and CLMBs

**Figure 3:**
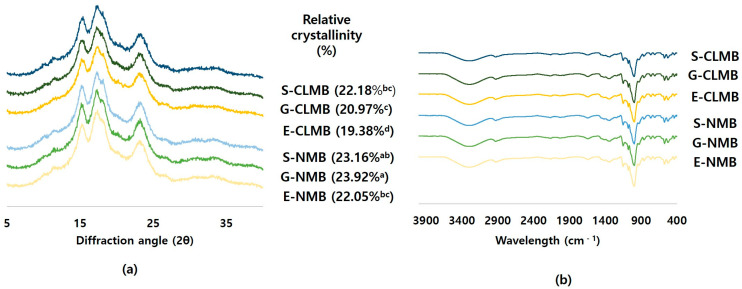
X-ray diffractogram patterns (**a**) and FT-IR spectra (**b**) of native mung bean starches (NMBs) and cross-linked mung bean starches (CLMBs) from three mung bean varieties: Eohul (E), Geunsung (G), and Sohyeon (S). The lowercase letters next to the relative crystallinity values represent significance groupings; values with the same letter are not significantly different.

The X-ray diffraction patterns of the NMBs and CLMB are presented in Figure 3. The NMBs exhibited main peaks at 2θ values of 15.1°, 17°, 18°, and 23°, which are characteristic of a typical A-type crystalline structure and consistent with those reported by No and Shin [13], Park et al. [24], and Huang et al. [27]. In contrast, legume starches have previously been reported to exhibit a C-type crystalline pattern with diffraction peaks at 5.5°, 7.0°, 18.0°, 20.0°, and 23.5° [30]. In MBs, variations in crystallinity patterns have been attributed to differences between cultivars, extraction methods, or X-ray analysis conditions [31].

The CLMBs retained their characteristic A-type crystalline structure, showing no notable differences compared to the NMBs. This aligns with the SEM results. Corn, rice, and tapioca starches also retain their crystalline structures, even after phosphorylation-induced cross-linking with STMP and STPP [4,9,11], which suggests that cross-linking with STMP and STPP primarily occurs in the amorphous regions of the starch granules. Notably, slight reductions in the intensity of specific diffraction peaks were noted in the CRMGBs when compared to their corresponding NMBs, potentially due to the substitution of hydroxyl groups with phosphate groups during the cross-linking process [9].

The relative crystallinity (RC) of the NMBs and CLMBs is shown in Figure 3. The RC of the NMBs varied between 22.05 and 23.92%, with G-NMB showing the highest value among the cultivars. After cross-linking, the RC values decreased, ranging from 19.38 to 22.18% (*p* < 0.05). Among varieties, RCs decreased slightly in G-CLMB and E-CLMB but showed no change in S-CLMB (*p* < 0.05). Since cross-linking primarily occurs in the amorphous regions of starch and does not disrupt the ordered crystalline structure, the RC typically remains unchanged or even increases [28]. The observed changes in the crystalline structure following cross-linking may be attributed to structural alterations in long-chain amylopectin or differences in processing conditions. For instance, in corn starch treated with STMP/STPP, a decrease in RC was reported, which was attributed to prolonged reaction times under high pH conditions during cross-linking under the slurry method [11]. During phosphorylation under high-moisture heat treatment, hydrogen bonds between molecular chains within starch granules are disrupted, leading to changes in the orientation, arrangement, compression, or structural integrity of starch double helices [29]. This weakens the crystalline structure, and the RC is reduced. Therefore, NMB treated with STMP/STPP may experience a reduction in crystalline structure due to phosphate groups with the interfering hydrogen bonding network during cross-linking treatment.

#### 3.1.5. FT-IR Spectra of NMBs and CLMBs

The FT-IR spectra of the NMBs and CLMBs, presented in Figure 3, reveal no significant differences among the mung bean cultivars; however, differences were observed between the NMBs and CLMBs. Both exhibited a peak at 3401 cm^−1^, attributed to the vibrational deformation of –OH groups [16]; at 1000 cm^−1^, indicating the presence of C6P–OH; and around 836–839 cm^−1^, attributed to the formation of P–O–C bonds [7,11]. However, no differences in the intensity of the vibrational bands were observed, which is presumed to be due to the esterification bonds already present within the starch structure [9]. The vibrational bands for O-H (1248 cm^−1^), C-H (1365–1413 cm^−1^), and C-O (1160 cm^−1^) displayed no notable differences between the NMBs and CLMBs, indicating that the fundamental chemical structures of both starches are largely unchanged. The overall similarity of the FT-IR spectra of the NMBs and CLMBs may indicate that their basic chemical structures are alike [4].

The degrees of order in the NMBs and CLMBs were estimated from the ratio of the intensities of the absorption bands at 1047 and 1022 cm^−1^ and the degree of double helix by calculating the 995:1022 cm^−1^ ratio (Table 1). The degrees of order of the CLMBs (0.67–0.70) were greater than those of the NMBs (0.62–0.66). Among the samples, G-CLMB exhibited the highest 1047:1022 cm^−1^ ratio. In a study on phosphorylated corn starch, the 1047:1022 cm^−1^ ratio significantly increased in phosphorylated starch treated with STMP or STPP [11]. These results indicate the successful formation of cross-links in the CLMBs, incorporating phosphate functional groups into their molecular structure.

#### 3.1.6. Water-Binding Capacity, Swelling Power, and Solubility of NMBs and CLMBs

The WBC of the NMBs and CLMBs is shown in Table 2. The unique thickening properties of starch arise from its ability to absorb water and swell, making its water-binding capacity a key index for starch products [29]. Starch primarily absorbs water into its amorphous regions or onto its surface, and the amount of absorbed water directly affects dough formation and processing suitability during food manufacturing [32]. All NMBs exhibited similar WBC values: 142.38% for S-NMB, 140.77% for E-NMB, and 138.72% for G-NMB. In contrast, the WBC of the CLMBs increased compared to those of the NMBs, with E-CLMB showing the highest WBC value (171.46%), followed by G-CLMB (157.56%) and S-CLMB (143.00%), all of which were significantly different from each other (*p* < 0.05). These increases can be attributed to the presence of hydrophilic phosphorus, which facilitates electrostatic repulsion between starch chains [33], reducing intermolecular bonding forces and enabling greater water absorption and swelling. The enhanced water-binding capacity suggests minor swelling in the amorphous regions and a disruption of hydrogen bonds connecting the crystalline and amorphous regions [30]. Previous studies have similarly reported that the introduction of negatively charged phosphate groups during phosphorylation enhances the WBC of STMP/STPP cross-linked starches by promoting water retention compared to native starch [4,9,33].

Swelling power and solubility serve as indicators for assessing the extent of interactions between starch chains in the amorphous and crystalline regions [3]. The swelling power and solubility of the NMBs and CLMBs, measured after heating at 80 °C, are presented in Table 2. Among the NMBs, E-NMB showed the highest swelling power (15.25 g/g), followed by S-NMB (12.10 g/g) and G-NMB (11.50 g/g), with significant differences identified between each value (*p* < 0.05). The cross-linked starches, E-CLMB (3.12 g/g), G-CLMB (3.47 g/g), and S-CLMB (2.90 g/g), all exhibited significantly lower swelling powers than the NMBs (*p* < 0.05). Swelling power represents the ability of starch granules to absorb water and expand at high temperatures [11]. This property is higher in native starches due to their relatively intact and flexible granular structure. Chung et al. [34] suggested that the reduction in swelling power can be attributed to two factors: (1) the transformation of amorphous amylose into a helical form and (2) stronger interactions occurring either between amylose chains or between amylose and amylopectin chains within the amorphous regions. The marked decrease in the swelling powers of the CLMBs may be explained by enhanced bonding between amylose and amylopectin molecules, which reduces leaching and promotes the formation of covalent bonds via phosphate groups. The cross-linking of starch increases both intra- and intermolecular interactions between amylose and amylopectin chains, thereby restricting the swelling of starch granules [35]. Additionally, the phosphorylation of starch also enhances the bonding between amylose and amylopectin, as well as preserving the structural integrity of starch granules by forming covalent bonds through phosphate groups, strengthening the granules, and making them more resistant to swelling [4]. Swelling resistance has been observed in phosphorylated corn starch [11].

The NMBs exhibited significantly higher solubilities (18.40% for E-NMB, 15.86% for G-NMB, and 15.98% for S-NMB) than the CLMBs (2.02% for S-CLMB, 1.34% for E-CLMB, and 1.22% for G-CLMB) (*p* < 0.05). Thus, E-NMB had the highest amylose content, which resulted in greater leaching of amylose during gelatinization. Solubility is influenced by the amylose content, with higher contents leading to increased solubility and the degree of interaction between starch chains [36]. Native starches have higher solubilities due to the leaching of amylose and partial disintegration of starch granules at high temperatures. Additionally, the diminished solubility in starch cross-linked with STMP/STPP may be attributed to the presence of phosphate groups, which facilitate the formation of additional covalent bonds between starch molecules [33].

#### 3.1.7. Thermal Properties of NMBs and CLMBs

The gelatinization onset temperature (T_o_), peak temperature (T_p_), conclusion temperature (T_c_), and gelatinization enthalpy (ΔH) values of the NMBs and CLMBs were in the range of 52.75–73.00 °C, 68.07–81.53 °C, 83.00–95.00 °C, and 9.49–15.84 J/g, respectively (Table 2). The lower T_o_ and T_c_ values observed in E-NMB suggest that its structure is more susceptible than those of the other MB varieties to disruption at lower temperatures. This aligns with the relatively high swelling power of E-NMB, as highly hydrated and swollen granules demand less energy to break the starch structure [1]. The other varieties, G-NMB and S-NMB, exhibited similar gelatinization temperatures, governed by the molecular structure of amylopectin, amylose content, relative crystallinity, or a combination of effects [22]. In this study, NMBs showed no significant differences in amylopectin branch chain length or relative crystallinity, which can be presumed to have contributed to the similar gelatinization temperatures observed.

In contrast, the CLMBs exhibited higher T_o_, T_p_, and T_c_ values than the NMBs. Improvement of the amylopectin crystal structure contributes to elevated thermal transition temperatures, enhancing the structural stability of starch granules and their resistance to gelatinization [19], and cross-linking further enhances the thermal stability of starch granules, enabling them to gelatinize at higher temperatures and maintain stability throughout the process [35]. Thus, the enhanced thermal stability seen in the CLMBs is likely a result of the formation of internal networks within the starch granules facilitated by the cross-linking process.

In the DSC analysis, the observed enthalpy primarily results from the disruption of the double helices rather than the disruption of the crystalline structure [21]. In most cases, higher ΔH values were observed in the CLMBs, confirming that more energy is required to disrupt their crystalline structures during melting. This is linked to the cross-linking modification, which reduces the ordered regions within the starch granules and enhances their resistance to digestion [37]. An increased ΔH also indicates a more complex energy transformation process during thermal treatment [38]. Previous studies have shown that the ΔH value increases with higher levels of cross-linking, which is attributed to the introduction of phosphate groups that result in a denser internal starch granule structure [35]. Notably, E-CLMB showed a lower ΔH than E-NMB, suggesting strong varietal differences, likely due to variations in the amylose/amylopectin ratios and degrees of molecular aggregation.

#### 3.1.8. In Vitro Digestibility of NMBs and CLMBs

The in vitro digestibility of the NMBs and CLMBs is shown in Table 3. The RDS, SDS, and RS values of the NMBs ranged from 24.53 to 24.16%, 16.69 to 21.49%, and 54.97 to 59.15%, respectively. Eohul’s NMB, which exhibited the highest swelling power, was found to have the lowest RS content, as highly swollen granules allow enzymes easier access to the starch granules [1]. In contrast, higher RS contents were observed in G-NMB and S-NMB, which also exhibited relatively lower swelling powers. In this experiment, S-NMB had a higher molecular weight of amylopectin than the other MB varieties, which is believed to have positively contributed to the increased RS content [26]. The susceptibility of starch to amylase digestion is affected by the presence of α-1, 6-linkages, and the proportion of linear amylopectin chains, as branched chains, exhibit greater resistance to enzymatic hydrolysis [16]. Therefore, differences in digestibility among varieties appear to be due to differences in the molecular structure of their starches.

Although differences exist among varieties, MB starches generally exhibit higher RS contents than other starch sources. This can be attributed to MBs’ natural starch structure, in which amorphous and crystalline regions are concentrically arranged, contributing to their high resistance to digestion [26]. Short amylopectin chains have difficulty forming double helices, making them more easily digestible. Higher molecular weights of amylopectin and greater amylose contents are associated with an increased RS content [26]. As a result, enzymatic sensitivity in starch is positively correlated with the proportion of amylopectin with DPs of 6–12 and negatively correlated with amylose [39]. In this experiment, NMB also exhibited a higher RS content than other native starches, likely due to its longer amylopectin chain length and higher amylose content. In Sandhu and Lim [21], a high RS content was observed in NMB, with SDS and RS contents reported as 40.0% and 50.4%, respectively. However, NMBs may undergo structural changes during heating, and the RS content of starch can vary depending on the cooking conditions, often showing a tendency to decrease during heat-based cooking [26]. Therefore, since the RS content of NMB may decrease upon heat treatment, its direct application as a functional ingredient is limited.

Compared to the NMBs, the CLMBs showed decreases in RDS (21.74–22.70%) and SDS (11.39–13.35%) and a significant increase in RS (63.95–66.87%) (*p* < 0.05). The incorporation of phosphate groups during phosphorylation appears to transform part of the rapidly digestible amorphous regions of native starch into RS4. Under alkaline conditions, STMP cross-linking can produce phosphodiester bonds between amylose and amylopectin, between amylopectins, or between starch molecules and surface-localized proteins [40]. This restricts the entry of α-amylase through porous channels into the interior of cereal starches [7]. The newly formed cross-links within the starch molecules significantly increase the RS content while reducing the SDS fraction, leading to a notable decrease in overall starch digestibility. Thus, STMP/STPP cross-linking treatments hold significant potential for producing low-GI starch, expanding its industrial applications in low-GI functional foods for diabetic and hyperglycemic patients [41]. A previous study reported that phosphorylated starch exhibits an increased bile salt-binding capacity in its cross-linked regions, suggesting it has the potential to facilitate bile acid excretion in the intestine [4]. Therefore, CLMB has the potential to be utilized as a food ingredient for increasing the RS content. To support this application, future studies should investigate how these resistant starch-containing cross-linked starches are digested in the human body.

### 3.2. Gel Properties and Freeze–Thaw Stability of NMB and CLMB Gels

#### 3.2.1. Appearance and Color Values of the NMB and CLMB Gels

The gelatinization process of starch involves the absorption of water and swelling of starch particles. When heated above a specific temperature, these particles lose their crystalline structure and transition into an amorphous state [27]. During the swelling process, amylose is leached from the starch granules and subsequently forms a three-dimensional network. Upon subsequent cooling, this network traps water within the granules, leading to the formation of a gel matrix structure. This process is crucial in starch-based processed foods, making it essential to understand the resulting gel’s properties. Due to its high amylose content, MB starch has remarkable gelation capabilities, which makes it a desirable ingredient in the food industry, especially for the production of noodles [1,2,3]. As a result, a thorough analysis of its gelation properties is necessary to determine its possible industrial uses and enhance its functional performance in a range of food applications. To evaluate the gel characteristics of the CLMBs and NMBs, gels were prepared, and their properties were analyzed.

**Figure 4:**
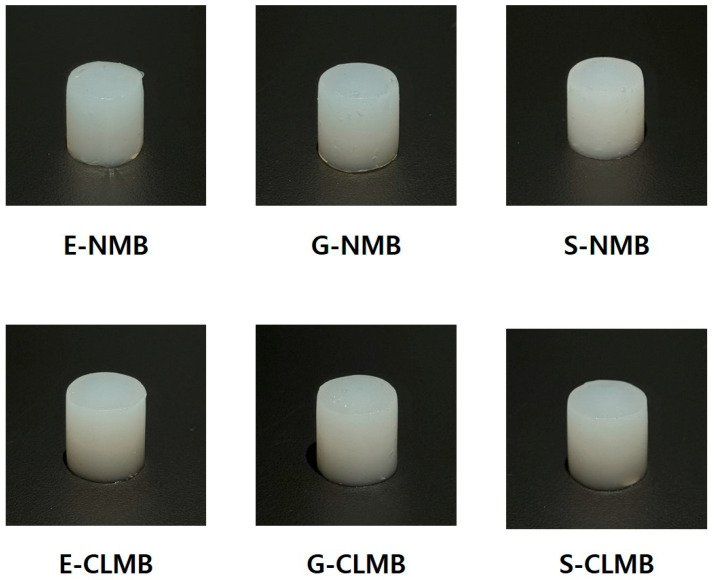
Appearance of gels derived from native mung bean starches (NMBs) and cross-linked mung bean starches (CLMBs) from three mung bean varieties: Eohul (E), Geunsung (G), and Sohyeon (S).

Figure 4 and Table 4 show the appearance and color values, respectively, of gels made with NMB alone and those including 10% CLMB. When gels were prepared using CLMB alone, they failed to form a stable gel structure. This instability is attributed to the high degree of cross-linking induced by phosphate-based covalent bonds, which restricts the swelling of starch granules and enhances their resistance to heating [4,11].

The NMBs and 10% CLMB mixtures formed firm gels, with no significant differences observed among the MB starches based on variety. However, the 10% CLMB gels appeared slightly more opaque than the NMB gels, which were created with starches exhibiting higher swelling powers. Previous studies have reported that phosphorylation with STMP/STPP reduces starch transmittance by inducing high levels of cross-linking, enhancing particle rigidity, and forming an opaque starch paste [11]. This rigidity reduced the swelling power and solubility, ultimately impacting the transmittance of the gel by reflecting light and diminishing paste clarity [4]. Therefore, greater opacity of the 10% CLMB gels indicates the successful formation of cross-linking in CLMB.

While differences in opacity were noted, very few significant differences were observed in the color values measured using a colorimeter. The colorimeter was optimized for measuring lightness, redness, and yellowness rather than transparency, which may lie outside the scope of the instrument’s capabilities. Additionally, according to Dhull et al. [5], the gelatinized products of modified starches exhibit minimal visual distinctions, resulting in negligible color differences. This suggests that the modification did not significantly impact the color of the NMB and 10% CLMB gels, and no significant differences were detected. Among the samples, the only significant color difference was seen in G-NMB, which exhibited a low L value (47.16), resulting in a significantly higher ΔE value. These differences are likely due to the structural characteristics of the starch.

#### 3.2.2. Textural Properties of NMB and CLMB Gels

The texture properties of NMB and 10% CLMB gels, both fresh and after 16 h of refrigeration, are presented in Table 5. The mechanical characteristics of the overall mung bean starch gel network can be influenced by the characteristics of the surrounding matrix and amylose swelling. The MB starch gels have previously been observed to be firm, transparent, elastic, and non-fragile [24]. The hardness values of freshly prepared gels were 274.14 g for E-NMB, 308.42 g for G-NMB, 398.71 g for S-NMB, 260.71 g for E-CLMB, 279.71 g for G-CLMB, and 291.00 g for S-CLMB. The textural properties of starch-based gels can be influenced by macromolecular additives due to the spacing effect and competitive water absorption [42]. Among the samples, E-NMB, which had the highest swelling power, exhibited the lowest hardness value. This suggests that the primary structural variable in starch gels is the deformability of swollen granules, with lower amylose leachability producing greater gel strength [1]. Therefore, the low hardness of E-NMB in this study can be attributed to its high solubility. The viscoelastic properties of starch gels depend significantly on amylopectin B3 chains and amylose contents, as longer chain lengths enhance amylopectin interactions and promote bond formation [23]. The starches of the MB varieties used in this study exhibited similar amylopectin chain length distributions, which resulted in minimal differences in gelling and viscoelastic properties.

Among the MB varieties, gel hardness was only affected by the STMP/STPP treatment in Geumsung, and 10% G-CLMB gel showed lower values than the G-NMB gel. This can be attributed to variations in amylose content and granule size, which are known to significantly influence texture properties [43]. Previous research has shown that STMP and STMP/STPP phosphorylation processes reduce amylose contents in starches due to cross-linking between amylose and amylopectin [11], which may have influenced the gel properties of the starch samples in this study.

The addition of CLMB significantly affected the texture and stability of the resulting gels, creating softer structures with lower hardness and chewiness compared to gels derived from only NMB [44]. Cohesiveness, which reflects the internal strength and structural integrity of the gel network, generally decreased in the 10% CLMB gels when compared to the corresponding NMB gels (*p* < 0.05). In RS4-modified samples, cohesiveness was slightly reduced, indicating a weaker but more flexible gel network [45]. This change is attributed to the formation of cross-links, which restricted the swelling of starch granules and increased their resistance to heat, ultimately influencing the gel network structure [11].

Springiness characterizes a gel’s ability to recover its shape after compression, while gumminess measures the energy required to break the semisolid gel into a swallowable state [42]. Springiness remained consistent across the NMB and 10% CLMB gels, whereas gumminess tended to be lower in CLMB-containing gels (200.28–237.42) than in NMB gels (220.57–250.00). Phosphorylation with STPP has been shown to significantly increase the tan δ value of the starch, indicating higher viscosity and lower elasticity [11]. This suggests that CLMB alone is less capable of forming a firm gel than NMB and exhibits stronger viscous properties, and when combined, these characteristics are believed to influence the texture properties of the resulting gel.

To assess the degree of retrogradation in starch gels, the prepared gels were stored in a refrigerator for 16 h, and their properties were analyzed (Table 5). Retrogradation causes amylose and amylopectin chains to realign and form stronger hydrogen bonds, which weakens the gel structure over time, decreasing hardness and chewiness [46]. Additionally, water migration during storage contributes to dehydration and shrinkage, further reducing gel integrity.

After 16 h of storage, the hardness and gumminess values of all starch gels increased, and the NMB gels showed significantly higher hardness and gumminess values than the 10% CLMB gels (*p* < 0.05). This increase in hardness is associated with the retrogradation of amylose and amylopectin in the starch [47]. Amylose gelation is considered a partial crystallization process, and the gel network of amylose forms relatively quickly and remains stable during storage. In contrast, the gelation of amylopectin within swollen granules occurs more slowly, resulting in an increase in gel hardness over time [24].

The increase in hardness due to retrogradation during storage is associated with the average chain length of amylopectin, as a higher proportion of longer chains may accelerate retrogradation [1]. Compared to other plant starches, MB starch exhibits a much higher tendency for retrogradation due to its high amylose content [19]. This suggests that retrogradation affects both amylose and amylopectin. In CLMB, as indicated by the reduction in relative crystallinity, the phosphate cross-linking process involves reactions with long amylopectin chains over a portion of their length, resulting in a loss of crystalline structure. This provides evidence that CLMB may contribute to the relative inhibition of retrogradation when incorporated into starch gels.

It has been reported that the high levels of cross-linking via covalent bonds involving phosphate groups restrict starch granule swelling and increase thermal resistance [11]. Cross-linking improves the structural integrity of starch, and studies have shown that the impact of hydrothermal treatment on starch structure is limited after phosphorylation [29]. This, in turn, enhances the entanglement of amylopectin, thereby strengthening the gel network [11]. Excessive cross-linking can decrease flexibility and result in a less firm gel structure, even while it strengthens the starch network by limiting swelling. In order to apply CLMB as a material that makes use of the gelation capabilities of mung bean starch, it is imperative that its utilization be appropriately adjusted. Furthermore, CLMB has a great chance of being used in food goods that need to be stored for a long time because it helps to prevent retrogradation.

#### 3.2.3. Freeze–Thaw Stability of NMB and CLMB Gels

The NMB and 10% CLMB gels were subjected to freeze–thaw tests (Table 6). Freeze–thaw stability, measured as the % liquid loss due to syneresis, was determined after the first, third, and fifth freeze–thaw cycles. When starch gels freeze, the formation of internal ice increases the starch concentration, and temperature fluctuations above the glass transition temperature during repeated freeze–thaw cycles promote the recrystallization of starch molecules [48]. During the thawing process, the melting of ice leads to the separation of water from the gel network, a phenomenon known as syneresis. Syneresis is undesirable in starch pastes and food products because it reduces paste stability and negatively affects product quality and shelf life [49]. Resistance to syneresis is a key indicator for evaluating the freeze–thaw stability of a starch gel [18].

During the first cycle, the syneresis of the NMB and 10% CLMB gels was measured at 6.81–8.55% and 4.70–5.85%, respectively. Across all MB varieties, the syneresis rates of gels made with CLMB were lower than those of NMB gels. In the third and fifth cycles, the gels released more water, with syneresis in the NMB gels ranging from 10.75 to 14.70% and 14.98 to 19.98%, respectively, while 10% CLMB gels showed syneresis rates of 9.07–9.86% and 10.87–13.73%, respectively (*p* < 0.05). These results indicate that the inclusion of CLMB effectively reduced syneresis, thereby enhancing freeze–thaw stability. The phosphorylation of starch reduces intramolecular hydrogen bonding, which is required to form the helical crystalline structure of starch, increasing solubility and water affinity [8]. The resulting densified starch structure appears to limit water migration during freeze–thaw cycles [29]. In waxy starches with a high proportion of amylopectin chains with DPs over 13, increasing STMP/STPP levels reportedly enhanced interactions between branched chains, accelerating retrogradation and increasing syneresis [49]. According to Li et al. [31], the swelling power of heat moisture treatment starch decreases due to the reorganization of structures within starch granules into more rigid forms. Similarly, the addition of CLMB is believed to have enhanced the freeze–thaw stability of the gels by forming a rigid internal structure through internal cross-linking. Han et al. [44] reported that including corn starch and compound phosphates in kuey teow-strengthened starch–water binding inhibited the conversion of free water into ice crystals and mitigated quality degradation caused by retrogradation. The incorporation of phosphate groups into the starch structure of CLMB increases its solubility and water-binding capacity, which helps it retain water in food products and contributes positively to its freeze–thaw stability.

Due to its remarkable freeze–thaw stability, CLMB is an ideal supplement to frozen foods, sauces, and ready-to-eat meals. It helps retain water during freeze–thaw cycles, preventing quality degradation due to its decreased syneresis and enhanced water-binding capacity. This feature is especially important in sauces and prepared foods that need to retain a consistent texture and stability, as well as for products that need to retain their nutritional value and texture, even after repeated freezing and thawing [50]. CLMB is a viable option for use in 3D printing and similar materials because of its regulated viscoelastic characteristics and enhanced freeze–thaw resilience. STMP/STPP cross-linked corn starch exhibited reductions in both G′ and G″ values, indicating a weakened starch paste network and decreased viscoelasticity, which resulted in the formation of gels with lower rigidity [11]. Materials with low viscosity and high self-supporting properties are the most commonly used printing substrates in 3D printer inks [51]. Consequently, CLMB’s capacity to gel while displaying decreased viscosity points to its possible use as a material for 3D printing and related processes.

### 3.3. Principal Component Analysis

**Figure 5:**
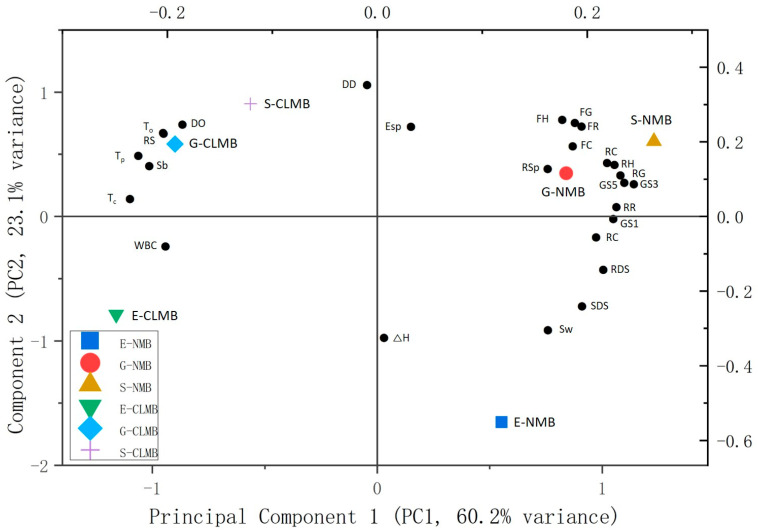
Principal component analysis (PCA) of native mung bean starches (NMBs) and cross-linked mung bean starches (CLMBs) from three mung bean varieties: Eohul (E), Geumsung (G), and Sohyeon (S). The variables incorporated into the PCA include swelling power (Sw); solubility (Sb); water-binding capacity (WBC); relative crystallinity (RC); the ratio of the FT-IR intensity at 1047 cm^−1^ to the intensity at 1022 cm^−1^ (DO); the ratio of the FT-IR intensity at 995 cm^−1^ to the intensity at 1022 cm^−1^ (DD); the proportions of rapidly digestible starch (RDS), slowly digestible starch (SDS), and resistant starch (RS); onset temperature (T_o_); peak temperature (T_p_); conclusion temperature (T_c_); enthalpy (ΔH); hardness (FH), resilience (FR), cohesiveness (FC), springiness (FSp), and gumminess (FG) of the freshly prepared gels; the hardness (RH), resilience (RR), cohesiveness (RC), springiness (RSp), and gumminess (RG) of the retrograded gels; and the one (GS1), three (GS3), and five (GS5) freeze–thaw cycle percent gel syneresis values.

The structure and physicochemical properties of the NMBs and CLMBs—including the degree of order, relative crystallinity, swelling power, solubility, water-binding capacity (WBC), in vitro digestibility, thermal and textural properties, and freeze–thaw stability—were analyzed using PCA, identifying two principal components, PC1 and PC2, which accounted for 60.2% and 23.1% of the variance, respectively. As illustrated in Figure 5, the NMBs (E-NMB, G-NMB, and S-NMB) were distinctly separated from the CLMBs (E-CLMB, G-CLMB, and S-CLMB) along the PC1 axis. This separation indicates that cross-linking significantly influenced the physicochemical characteristics of the starches.

Principal component 1 was primarily correlated with relative crystallinity, enthalpy (ΔH), and the proportions of RDS and SDS, while PC2 was predominantly associated with the degree of order, T_c_, WBC, swelling power, and the proportion of RS. The influence of the degree of order on the physicochemical properties of the starches, including RS content, indicates that effective structural changes were induced by cross-linking, and the other associated physicochemical properties suggest that the CLMBs exhibited lower swelling power and reduced digestibility, whereas the NMBs displayed higher solubility and greater swelling power. These findings align with those of a previous study, which reported positive correlations between swelling power, transmittance, peak viscosity, amylose content, solubility, relative crystallinity, gelatinization enthalpy, and T_o_ in corn starch treated with STMP or STMP/STPP [11].

In the current study, E-NMB differed from G-NMB and S-NMB in swelling power, while E-CLMB differed from the other CLMBs in WBC. These characteristics are closely related to the structural properties of starch, suggesting that understanding the amylose and amylopectin structures of MB varieties could enable the prediction of these quality attributes.

Overall, the analysis demonstrates that the treatment method significantly impacted the physicochemical properties of starch. Moreover, the results from the PCA were consistent with the theoretical analysis conducted in the study.

This study examined the structure and gelling properties of MB starches, and based on our results, CLMB demonstrates significant potential as a food ingredient with enhanced nutritional functionality due to its increased RS content. Additionally, it offers improved freeze–thaw stability and structural integrity. The native starches from different MB varieties exhibit structural variations, and when comparing them to the cross-linked starches, distinct differences in quality characteristics were observed. This suggests that understanding the structure of the starch could enable the prediction of its corresponding changes in physical properties during cross-linking treatment. While MB starch has the advantage of a high gel-forming ability, it is hindered by a high tendency for retrogradation. Cross-linking has been shown to improve the physical properties of NMB in this regard. Therefore, its structural integrity and functional properties indicate that CLMB could serve as a valuable material for advanced applications, such as in 3D printer inks or bio-films, warranting further rheological study.

## 4. Conclusions

This study aimed to produce CLMB using STMP and STPP and evaluate its structural characteristics, in vitro digestibility, gel stability, and freeze–thaw stability. The CLMB maintained an A-type crystalline structure, forming an ordered structural domain through phosphate cross-linking. As a result, its swelling power and solubility decreased, while its gelatinization temperature range increased. Additionally, the CLMB demonstrated reductions in RDS and SDS contents with a significant increase in RS content, indicating functional improvements. The CLMB also exhibited excellent gel stability and freeze–thaw stability, making it a suitable material for various food industry applications. In conclusion, cross-linked MB starch demonstrates potential as a promising ingredient for health-oriented and processed foods due to its reduced digestibility and improved retrogradation stability, highlighting its suitability for innovative food product development. Future research should prioritize the optimization of cross-linking conditions to further enhance the functional properties of CLMB, investigate the molecular mechanisms underlying gelation improvements, and assess its long-term health benefits through in vivo studies. Furthermore, the potential applications of CLMB in non-food industries, including 3D printer inks, biofilm production and drug delivery systems, merit further exploration.

## Figures and Tables

**Table 1:** FT-IR molecular orders of the native mung bean starches (NMBs) and cross-linked mung bean starches (CLMBs).

	R1047/1022	R995/1022
E-NMB	0.62 ± 0.00 ^f^	1.21 ± 0.01 ^b^
G-NMB	0.66 ± 0.00 ^d^	1.30 ± 0.02 ^a^
S-NMB	0.64 ± 0.00 ^e^	1.29 ± 0.02 ^a^
E-CLMB	0.67 ± 0.00 ^c^*	1.27 ± 0.02 ^a^
G-CLMB	0.70 ± 0.00 ^a^*	1.29 ± 0.02 ^a^
S-CLMB	0.68 ± 0.00 ^b^*	1.28 ± 0.02 ^a^

E-, G-, and S- indicate starches from the Eohul, Geumsung, and Sohyeon mung bean varieties. Data represent means ± SDs. ^a–f^ Values in the same column with different letters are significantly different (*p* < 0.05), as determined by Duncan’s multiple range tests. * CLMB values are significantly different from the corresponding NMBs, as determined using independent *t*-tests (*p* < 0.05).

**Table 2:** Physicochemical and thermal properties of native mung bean starches (NMBs) and cross-linked mung bean starches (CLMBs).

	Water-Binding Capacity (%)	Swelling Power at 80 °C (g/g)	Solubility at 80 °C (%)	Thermal Properties (Based on DSC)			
				Onset (T_o_)	Peak (T_P_)	Conclusion (T_c_)	Enthalpy (ΔH, J/g)
E-NMB	140.77 ± 4.19 ^c^	15.25 ± 0.07 ^a^	18.40 ± 0.28 ^a^	52.75 ± 1.77 ^d^	68.07 ± 1.12 ^d^	87.75 ± 1.77 ^bc^	15.84 ± 0.74 ^a^
G-NMB	138.72 ± 0.33 ^c^	11.50 ± 0.21 ^c^	15.86 ± 0.08 ^b^	60.50 ± 0.71 ^c^	70.48 ± 0.21 ^c^	87.00 ± 5.66 ^bc^	9.49 ± 0.46 ^c^
S-NMB	142.38 ± 0.51 ^c^	12.10 ± 0.07 ^b^	15.98 ± 0.03 ^b^	61.00 ± 0.00 ^c^	69.77 ± 0.03 ^c^	83.00 ± 0.00 ^c^	9.77 ± 0.22 ^c^
E-CLMB	171.46 ± 3.97 ^a^	3.12 ± 0.03 ^d^	1.34 ± 0.25 ^d^	71.00 ± 0.00 ^b^	79.32 ± 0.01 ^b^	93.00 ± 0.00 ^ab^	10.98 ± 0.04 ^b^
G-CLMB	157.56 ± 0.96 ^b^	3.47 ± 0.50 ^d^	1.22 ± 0.37 ^d^	72.00 ± 0.00 ^ab^	80.71 ± 0.04 ^a^	94.00 ± 0.00 ^a^	11.48 ± 0.41 ^b^
S-CLMB	143.00 ± 2.78 ^c^	2.90 ± 0.17 ^d^	2.02 ± 0.31 ^c^	73.00 ± 0.00 ^a^	81.53 ± 0.10 ^a^	95.00 ± 0.00 ^a^	11.17 ± 0.31 ^b^

E-, G-, and S- indicate starches from the Eohul, Geumsung, and Sohyeon mung bean varieties. Data represent means ± SDs. ^a–d^ Values in the same column with different letters are significantly different (*p* < 0.05), as determined by Duncan’s multiple range tests.

**Table 3:** In vitro digestibility of native mung bean starches (NMBs) and cross-linked mung bean starches (CLMBs).

	In Vitro Digestibility		
	RDS (%)	SDS (%)	RS (%)
E-NMB	23.53 ± 0.12 ^ab^	21.49 ± 0.53 ^a^	54.97 ± 0.65 ^e^
G-NMB	23.33 ± 0.89 ^ab^	19.20 ± 0.47 ^b^	57.48 ± 0.41 ^d^
S-NMB	24.16 ± 0.06 ^a^	16.69 ± 0.35 ^c^	59.15 ± 0.30 ^c^
E-CLMB	22.70 ± 0.59 ^bc^	13.35 ± 0.12 ^d^	63.95 ± 0.47 ^b^
G-CLMB	21.82 ± 0.18 ^c^	12.35 ± 0.59 ^de^	65.82 ± 0.77 ^a^
S-CLMB	21.74 ± 0.18 ^c^	11.39 ± 0.77 ^e^	66.87 ± 0.59 ^a^

The mean proportions (±SD) of the total starch content in the rapidly digestible starch (RDS), slowly digestible starch (SDS), and resistant starch (RS) categories. E-, G-, and S- indicate starches from the Eohul, Geumsung, and Sohyeon mung bean varieties. Data represent means ± SDs. ^a–e^ Values in the same column with different letters are significantly different (*p* < 0.05), as determined by Duncan’s multiple range tests.

**Table 4:** Color values of native mung bean starch (NMB) and 10% cross-linked mung bean starch (CLMB) gels.

	L	a	b	∆E
E-NMB	50.60 ± 0.80 ^a^	0.36 ± 0.75 ^a^	−9.92 ± 1.78 ^a^	50.46 ± 0.59 ^b^
G-NMB	47.16 ± 1.23 ^b^	−1.02 ± 1.25 ^a^	−12.56 ± 1.56 ^a^	54.38 ± 0.90 ^a^
S-NMB	51.78 ± 2.65 ^a^	0.92 ± 0.70 ^a^	−12.14 ± 5.72 ^a^	50.24 ± 1.83 ^b^
E-CLMB	51.28 ± 2.49 ^a^	−0.42 ± 1.60 ^a^	−9.26 ± 4.00 ^a^	49.90 ± 2.76 ^b^
G-CLMB	50.18 ± 1.73 ^ab^	−0.42 ± 0.89 ^a^	−9.90 ± 2.96 ^a^	50.94 ± 1.95 ^b^
S-CLMB	51.02 ± 1.74 ^a^	−0.44 ± 0.88 ^a^	−9.60 ± 2.00 ^a^	49.98 ± 1.74 ^b^

E-, G-, and S- indicate starches from the Eohul, Geumsung, and Sohyeon mung bean varieties. Data represent means ± SDs. ^a,b^ Values in the same column with different letters are significantly different (*p* < 0.05), as determined by Duncan’s multiple range tests.

**Table 5:** Textural properties of both freshly prepared and retrograded (after 16 h of refrigeration) native mung bean starch (NMB) and cross-linked mung bean starch (CLMB) gels.

		**Hardness** **(g)**	**Resilience**	**Cohesiveness**	**Springiness**	**Gumminess**
Freshly prepared gels	E-NMB	274.14 ± 5.22 ^de^	0.64 ± 0.01 ^c^	0.80 ± 0.01 ^ab^	0.97 ± 0.01 ^a^	220.57 ± 7.06 ^b^
	G-NMB	308.42 ± 12.53 ^a^	0.67 ± 0.01 ^ab^	0.80 ± 0.02 ^ab^	0.97 ± 0.01 ^a^	250.00 ± 14.00 ^a^
	S-NMB	298.71 ± 8.81 ^ab^	0.68 ± 0.01 ^a^	0.82 ± 0.01 ^a^	0.98 ± 0.01 ^a^	247.42 ± 7.18 ^a^
	E-CLMB	260.71 ± 13.18 ^e^	0.60 ± 0.01 ^d^	0.76 ± 0.02 ^c^	0.97 ± 0.01 ^a^	200.28 ± 11.87 ^c^
	G-CLMB	279.71 ± 10.32 ^cd^	0.64 ± 0.01 ^c^	0.79 ± 0.01 ^b^	0.98 ± 0.01 ^a^	222.71 ± 9.75 ^b^
	S-CLMB	291.00 ± 7.14 ^bc^	0.66 ± 0.01 ^b^	0.81 ± 0.02 ^ab^	0.98 ± 0.01 ^a^	237.42 ± 10.93 ^ab^
		**Hardness** **(g)**	**Resilience**	**Cohesiveness**	**Springiness**	**Gumminess**
Retrograded gels	E-NMB	703.00 ± 42.66 ^c^	0.68 ± 0.04 ^b^	0.79 ± 0.05 ^a^	0.96 ± 0.01 ^ab^	560.66 ± 64.22 ^bc^
	G-NMB	863.33 ± 7.55 ^b^	0.66 ± 0 ^ab^	0.75 ± 0.01 ^a^	0.97 ± 0.01 ^ab^	654.33 ± 19.55 ^b^
	S-NMB	969.33 ± 63.77 ^a^	0.70 ± 0.05 ^a^	0.81 ± 0.06 ^a^	0.99 ± 0.01 ^a^	794.66 ± 117.55 ^a^
	E-CLMB	600.33 ± 5.77 ^d^	0.60 ± 0.01 ^b^	0.73 ± 0.01 ^a^	0.96 ± 0.01 ^ab^	438.00 ± 4.66 ^c^
	G-CLMB	685.00 ± 4.66 ^c^	0.64 ± 0.01 ^ab^	0.74 ± 0.01 ^a^	0.97 ± 0.01 ^ab^	509.66 ± 13.11 ^c^
	S-CLMB	655.66 ± 12.88 ^cd^	0.64 ± 0.01 ^ab^	0.75 ± 0.01 ^a^	0.95 ± 0.01 ^b^	494.33 ± 13.77 ^c^

E-, G-, and S- indicate starches from the Eohul, Geumsung, and Sohyeon mung bean varieties. Data represent means ± SDs. ^a–e^ Values in the same column with different letters are significantly different (*p* < 0.05), as determined by Duncan’s multiple range tests.

**Table 6:** Freeze–thaw stability of native mung bean starch (NMB) and cross-linked mung bean starch (CLMB) gels.

		Gel Syneresis (%)	
	One Cycle	Three Cycles	Five Cycles
E-NMB	6.81 ± 1.87 ^ab^	10.75 ± 2.80 ^ab^	14.98 ± 4.58 ^ab^
G-NMB	8.55 ± 1.02 ^a^	13.37 ± 2.21 ^ab^	17.68 ± 5.47 ^ab^
S-NMB	7.38 ± 1.40 ^ab^	14.70 ± 3.11 ^a^	19.98 ± 3.63 ^a^
E-CLMB	5.41 ± 1.13 ^b^	9.24 ± 2.77 ^b^	10.87 ± 2.89 ^b^
G-CLMB	4.70 ± 0.48 ^b^	9.07 ± 1.95 ^b^	11.34 ± 1.89 ^b^
S-CLMB	5.85 ± 1.29 ^b^	9.86 ± 1.78 ^ab^	13.73 ± 1.75 ^ab^

E-, G-, and S- indicate starches from the Eohul, Geumsung, and Sohyeon mung bean varieties. Data represent means ± SDs. ^a,b^ Values in the same column with different letters are significantly different (*p* < 0.05), as determined by Duncan’s multiple range tests.

## Data Availability

The original contributions presented in the study are included in the article; further inquiries can be directed to the corresponding author.

## Abbreviations

The following abbreviations are used in this manuscript:

MB	Mung bean
NMB	Native mung bean starch
CLMB	Cross-linked mung bean starch
E	Eohul
G	Geumsung
S	Sohyeon
STMP	Sodium trimetaphosphate
STPP	Sodium tripolyphosphate
GOPOD	Glucose oxidase/peroxidase
NaOH	Sodium hydroxide
HCl	Hydrochloric acid
Na_2_SO_4_	Sodium sulfate
SEM	Scanning electron microscope
DP	Degree of polymerization
RDS	Rapidly digestible starch
SDS	Slowly digestible starch
RS	Resistant starch
RC	Relative crystallinity
WBC	Water-binding capacity
T_o_	Onset temperature
T_p_	Peak temperature
T_c_	Conclusion temperature

## References

[1] Gunaratne A., Gan R., Wu K., Kong X., Collado L., Arachchi L.V., Kumara K., Pathirana S.M., Corke H. (2018). Physicochemical Properties of Mung Bean Starches Isolated from Four Varieties Grown in Sri Lanka. Starch-Stärke.

[2] Carrero-Carralero C., Mansukhani D., Ruiz-Matute A.I., Martínez-Castro I., Ramos L., Sanz M.L. (2018). Extraction and Characterization of Low Molecular Weight Bioactive Carbohydrates from Mung Bean (*Vigna radiata*). Food Chem..

[3] Li W., Guo H., Wang P., Tian X., Zhang W., Saleh A.S., Zheng J., Ouyang S., Luo Q., Zhang G. (2015). Physicochemical Characteristics of High-Pressure Gelatinized Mung Bean Starch During Recrystallization. Carbohydr. Polym..

[4] Ashwar B.A., Gani A., Shah A., Masoodi F.A. (2017). Physicochemical Properties, In-Vitro Digestibility and Structural Elucidation of RS4 from Rice Starch. Int. J. Biol. Macromol..

[5] Dhull S.B., Tanwar M., Khatkar S.K., Chandak A., Chawla P., Goksen G. (2024). Exploring the Effects of Thermal and Non-Thermal Modification Methods on Morphological, Functional, and Pasting Properties of Mung Bean Starch. Innov. Food Sci. Emerg. Technol..

[6] Shukri R., Shi Y.C. (2015). Physiochemical Properties of Highly Cross-Linked Maize Starches and Their Enzymatic Digestibilities by Three Analytical Methods. J. Cereal Sci..

[7] Dong H., Vasanthan T. (2020). Amylase Resistance of Corn, Faba Bean, and Field Pea Starches as Influenced by Three Different Phosphorylation (Cross-Linking) Techniques. Food Hydrocoll..

[8] Ramadan M.F., Sitohy M.Z. (2020). Phosphorylated Starches: Preparation, Properties, Functionality, and Techno-Applications. Starch-Stärke.

[9] Zhao Y., Zheng Z., Zhao Y., Chen J., Tang S. (2024). Cross-Linked Modification of Tapioca Starch by Sodium Trimetaphosphate: An Influence on Its Structure. Food Chem. X.

[10] Sha X., Gu Z., Zhang F., Jiang H. (2025). Exploring Characteristics of Instant Fried Noodles Enriched with Cross-Linked Phosphorylated Type 4 Resistant Wheat Starch: Insights from Its Microstructure, Textural Properties, and In-Vitro Starch Digestibility. Food Res. Int..

[11] Ma L., Liu J., Cheng Y., Frank J., Liang J. (2025). Structural Features, Physiological Functions and Digestive Properties of Phosphorylated Corn Starch: A Comparative Study of Four Phosphorylating Agents and Two Preparation Methods. Int. J. Biol. Macromol..

[12] Lemos P.V.F., Opretzka L.C.F., Almeida L.S., Cardoso L.G., Da Silva J.B.A., De Souza C.O., Villarreal C.F., Druzian J.I. (2020). Preparation and Characterization of C-Phycocyanin Coated with STMP/STPP Cross-Linked Starches from Different Botanical Sources. Int. J. Biol. Macromol..

[13] No J., Shin M. (2016). Textural Properties of Mung Bean Starch Gels Prepared from Whole Seeds. Food Sci. Biotechnol..

[14] Song J., Park J.H., Shin M. (2011). The effects of annealing and acid hydrolysis on resistant starch level and the properties of cross-linked RS4 rice starch. Starch-Stärke.

[15] Oh S., Shin M. (2015). Physicochemical Properties and Molecular Structures of Korean Waxy Rice Starches. Food Sci. Biotechnol..

[16] No J., Shin M. (2023). Structures and Digestibility of B-Type High-Amylose Rice Starches Compared with A-Type High-Amylose Rice Starches. J. Cereal Sci..

[17] Chel-Guerrero L., Barbosa-Martín E., Martínez-Antonio A., González-Mondragón E., Betancur-Ancona D. (2016). Some physicochemical and rheological properties of starch isolated from avocado seed. Int. J. Biol. Macromol..

[18] Park H.J., Cho D.H., Chung H.J., Lim S.T. (2024). Enhanced Gelling Property and Freeze-Thaw Stability of Potato, Tapioca, and Corn Starches Modified by Mild Heating in Aqueous Ethanol Solution. J. Sci. Food Agric..

[19] Kim S.H., Lee B.H., Baik M.Y., Joo M.H., Yoo S.H. (2007). Chemical Structure and Physical Properties of Mung Bean Starches Isolated from Five Domestic Cultivars. J. Food Sci..

[20] Israkarn K., Bakornpanom N.N., Hongsprabhas P. (2014). Physicochemical Properties of Starches and Proteins in Alkali-Treated Mungbean and Cassava Starch Granules. Carbohydr. Polym..

[21] Sandhu K.S., Lim S.T. (2008). Digestibility of Legume Starches as Influenced by Their Physical and Structural Properties. Carbohydr. Polym..

[22] Kim Y.Y., Woo K.S., Chung H.J. (2018). Starch Characteristics of Cowpea and Mung Bean Cultivars Grown in Korea. Food Chem..

[23] Qiao J., Jia M., Niu J., Zhang Z., Xing B., Liang Y., Li H., Zhang Y., Ren G., Qin P. (2024). Amylopectin Chain Length Distributions and Amylose Content Are Determinants of Viscoelasticity and Digestibility Differences in Mung Bean Starch and Proso Millet Starch. Int. J. Biol. Macromol..

[24] Park S.J., Choe E.O., Kim J.I., Shin M. (2012). Physicochemical Properties of Mung Bean Starches in Different Korean Varieties and Their Gel Textures. Food Sci. Biotechnol..

[25] Hongsprabhas P. (2007). On the Gelation of Mung Bean Starch and Its Composites. Int. J. Food Sci. Technol..

[26] Ma M., Wang Y., Wang M., Jane J.L., Du S.K. (2017). Physicochemical Properties and In Vitro Digestibility of Legume Starches. Food Hydrocoll..

[27] Huang Z., Li Y., Guo T., Xu L., Yuan J., Li Z., Yi C. (2024). The Physicochemical Properties and Structure of Mung Bean Starch Fermented by *Lactobacillus plantarum*. Foods.

[28] Ma M., Xu Z., Wu H., Li K., Sun G., He J., Sui Z., Corke H. (2022). Removal of Starch Granule-Associated Surface and Channel Lipids Alters the Properties of Sodium Trimetaphosphate Crosslinked Maize Starch. Int. J. Biol. Macromol..

[29] Huang P.H., Chiu C.S., Chan Y.J., Su W.C., Wang C.C.R., Lu W.C., Li P.H. (2024). Effect of Osmotic Pressure and Simultaneous Heat-Moisture Phosphorylation Treatments on the Physicochemical Properties of Mung Bean, Water Caltrop, and Corn Starches. Int. J. Biol. Macromol..

[30] Gani A., Jan A., Shah A., Masoodi F.A., Ahmad M., Ashwar B.A., Akhter R., Wani I.A. (2016). Physico-Chemical, Functional, and Structural Properties of RS3/RS4 from Kidney Bean (*Phaseolus vulgaris*) Cultivars. Int. J. Biol. Macromol..

[31] Li S., Gao Q., Ward R. (2011). Physicochemical Properties and In Vitro Digestibility of Resistant Starch from Mung Bean (*Phaseolus radiatus*) Starch. Starch-Stärke.

[32] Yoo J.S., Park H.S., Cho Y.C., Kim B.K., Ha K.Y. (2013). Comparison of Physicochemical and Textural Properties of Glutinous Rice Cultivars. Food Eng. Prog..

[33] Bangar S.P., Sunooj K.V., Navaf M., Phimolsiripol Y., Whiteside W.S. (2024). Recent Advancements in Cross-Linked Starches for Food Applications—A Review. Int. J. Food Prop..

[34] Chung K.M., Moon T.W., Chun J.K. (2000). Influence of Annealing on Gel Properties of Mung Bean Starch. Cereal Chem..

[35] Sharma V., Kaur M., Sandhu K.S., Godara S.K. (2020). Effect of Cross-Linking on Physicochemical, Thermal, Pasting, In Vitro Digestibility, and Film-Forming Properties of Faba Bean (*Vicia faba* L.) Starch. Int. J. Biol. Macromol..

[36] Cai W.C.J., Man J.M., Huang J.J., Liu Q.Q., Wei W.W., Wei C.X. (2015). Relationship Between Structure and Functional Properties of Normal Rice Starches with Different Amylose Contents. Carbohydr. Polym..

[37] Song J.Y., Lee J., No J. (2024). Physicochemical Properties and Digestibility of Rice Starch-Protein Complexes Treated with Transglutaminase. Food Sci. Technol..

[38] Nguyen T.L., Mitra S., Gilbert R.G., Gidley M.J., Fox G.P. (2019). Influence of Heat Treatment on Starch Structure and Physicochemical Properties of Oats. J. Cereal Sci..

[39] Lin L., Gu D., Huang J., Zhang X., Zhang L., Wei C. (2016). Molecular structure and enzymatic hydrolysis properties of starches from high-amylose maize inbred lines and their hybrids. Food Hydrocoll..

[40] Shin M., Song J., Seib P.A. (2024). In vitro Digestibility of Cross-Linked Starches–RS4. Starch-Stärke.

[41] Bodjrenou D.M., Huang Z., Liu T., Zheng B., Zeng H. (2023). Effects of Crosslinking with Sodium Trimetaphosphate on Structural, Physicochemical, Rheological and In Vitro Digestibility Properties of Purple Sweet Potato Starch. Food Res. Int..

[42] Min C., Ma W., Kuang J., Huang J., Xiong Y.L. (2022). Textural Properties, Microstructure and Digestibility of Mung Bean Starch–Flaxseed Protein Composite Gels. Food Hydrocoll..

[43] Singh N., Singh J., Kaur L., Sodhi N.S., Gill B.S. (2003). Morphological, Thermal and Rheological Properties of Starches from Different Botanical Sources. Food Chem..

[44] Han J., Wu J., Liu X., Shi J., Xu J. (2023). Physiological Effects of Resistant Starch and Its Applications in Food: A Review. Food Prod. Process. Nutr..

[45] Yu M., Shin M. (2015). Improving Gel Formation of Rice Starch Added with Cross-Linked Resistant Starch Prepared from Rice Starch. Starch-Stärke.

[46] Köksel H., Basman A., Kahraman K., Ozturk S. (2007). Effect of Acid Modification and Heat Treatments on Resistant Starch Formation and Functional Properties of Corn Starch. Int. J. Food Prop..

[47] Li G., Zhu F. (2018). Quinoa Starch: Structure, Properties, and Applications. Carbohydr. Polym..

[48] Srichuwong S., Isono N., Jiang H., Mishima T., Hisamatsu M. (2012). Freeze–Thaw Stability of Starches from Different Botanical Sources: Correlation with Structural Features. Carbohydr. Polym..

[49] Gu Z., Sha X., Wang X., Zhao R., Khashaba R., Jane J.L., Jiang H. (2024). Exploring characteristics of waxy wheat starches cross-linked at low levels: Insights from pasting, rheological, and textural properties. Food Biosci..

[50] Lee J.H., Kim M.J., Kim C.Y. (2024). The Development of New Functional Foods and Ingredients. Foods.

[51] Cheng Y., Yuqing H., Xiao L., Gao W., Kang X., Sui J., Cui B. (2024). Impact of Starch Amylose and Amylopectin on the Rheological and 3D Printing Properties of Corn Starch. Int. J. Biol. Macromol..

